# Parallel Changes in Mood and Melatonin Rhythm Following an Adjunctive Multimodal Chronobiological Intervention With Agomelatine in People With Depression: A Proof of Concept Open Label Study

**DOI:** 10.3389/fpsyt.2018.00624

**Published:** 2018-12-11

**Authors:** Rebecca Robillard, Joanne S. Carpenter, Kristy-Lee Feilds, Daniel F. Hermens, Django White, Sharon L. Naismith, Delwyn Bartlett, Bradley Whitwell, James Southan, Elizabeth M. Scott, Ian B. Hickie

**Affiliations:** ^1^Sleep Research Unit, The Royal Institute for Mental Health Research, Ottawa, ON, Canada; ^2^School of Psychology, Faculty of Social Sciences, University of Ottawa, Ottawa, ON, Canada; ^3^Clinical Research Unit, Brain & Mind Centre, The University of Sydney, Camperdown, NSW, Australia; ^4^Sunshine Coast Mind and Neuroscience–Thompson Institute, University of the Sunshine Coast, Sunshine Coast, QLD, Australia; ^5^Healthy Brain Ageing Program, Faculty of Science, Charles Perkins Centre, The University of Sydney, Sydney, NSW, Australia; ^6^Woolcock Institute of Medical Research, Sydney, NSW, Australia

**Keywords:** melatonin agonist, agomelatine, melatonin, depression, circadian rhythms dysfunctions

## Abstract

**Background:** Agomelatine is a melatonin agonist and 5HT antagonist developed for the treatment of major depressive disorder which also has some effects on the circadian system. Since circadian dysfunctions are thought to play a role in the pathophysiology of depression, some of the mechanism of action of this drug may relate to improvements in circadian rhythms.

**Objective:** This proof of concept open-label study sought to determine if improvements in depressive symptoms following an adjunctive multimodal intervention including agomelatine intake are associated with the magnitude of circadian realignment. This was investigated in young people with depression, a subgroup known to have high rates of delayed circadian rhythms.

**Methods:** Young people with depression received a psychoeducation session about sleep and circadian rhythms, were asked to progressively phase advance their wake up time, and completed an 8 weeks course of agomelatine (25–50 mg). Participants underwent semi-structured psychological assessments, ambulatory sleep-wake monitoring and measurement of melatonin circadian phase before and after the intervention.

**Results:** Twenty-four young adults with depression (17–28 years old; 58% females) completed the study. After the intervention, depressive symptoms were significantly reduced [*t*_(23)_ = 6.9, *p* < 0.001] and, on average, the timing of dim light melatonin onset (DLMO) shifted 3.6 h earlier [*t*_(18)_ = 4.4, *p* < 0.001]. On average, sleep onset was phase shifted 28 min earlier [*t*_(19)_ = 2.1, *p* = 0.047] and total sleep time increased by 24 min [*t*_(19)_ = –2.6, *p* = 0.018]. There was no significant change in wake-up times. A strong correlation (*r* = 0.69, *p* = 0.001) was found between the relative improvements in depression severity and the degree of phase shift in DLMO.

**Conclusion:** Although this needs to be replicated in larger randomized controlled trials, these findings suggest that the degree of antidepressant response to a multimodal intervention including psychoeducation and agomelatine intake may be associated with the degree of change in evening melatonin release in young people with depression. This offers promising avenues for targeted treatment based on the prior identification of objective individual characteristics.

## Introduction

In the ongoing search for more effective depression treatments, the last decade has seen the emergence of new melatonergic agents developed to improve mood (1). For instance, agomelatine, licensed as an antidepressant in Europe and Australia, is a first-in-class melatonin agonist at MT1 and MT2 receptors, and a 5HT antagonist at 5-HT2b and 5-HT2c receptors. The antidepressant response to agomelatine has been found to be superior to placebo (2–4), and large open-label studies in clinical settings are supportive of agomelatine's utility (5, 6). However, the magnitude of mood changes following agomelatine intake has been questioned (7–10). Of the two latest meta-analyses covering both published and unpublished findings about this drug, one concluded that the overall effect size of the antidepressant effects of agomelatine is of doubtful clinical significance (2), while the other emphasized that the efficacy of agomelatine is comparable to standard antidepressant drugs (4). One of the limitations of previous large clinical trials is the inclusion of widely heterogeneous samples of people with depression, most likely with a broad range of pathophysiological phenotypes. Due to its melatonergic pharmacological profile, agomelatine may be optimally suited to treat depression in individuals with prominent disruptions in circadian rhythms, a factor increasingly recognized as contributing to the onset and maintenance of depression for at least a subgroup of people with mood disorders. In order to identify when agomelatine is best indicated for the treatment of depression, a better understanding of the biological mechanisms underpinning the antidepressant effects of this drug is required.

Experimental work in healthy individuals has demonstrated that altering the timing of the sleep-wake cycle, or that of endogenous circadian rhythms, can have a significant influence on mood (11, 12). It has long been proposed that circadian disruptions may play a role in the pathophysiology of mood disorders (13–19), and that resynchronizing the biological clock may alleviate depressive symptoms (20–26). Agomelatine targets the melatonin system, which closely interacts with the biological clock, inducing robust circadian phase shifts (27–29). Hence, part of its antidepressant effects may operate via the resynchronization of circadian rhythms. Several theories about the mechanism of action underlying the antidepressant effects of melatonin agonists have been proposed, but the human empirical data on this topic remain scarce.

Diverse profiles of endogenous melatonin secretion have been linked to depression, with reports of both advanced (30) and delayed (31, 32) circadian phase. Age may play a considerable role in this heterogeneity. A circadian phase-delay normally occurs during adolescence and young adulthood (33, 34), and our recent work suggests that delays in the sleep-wake cycle (35, 36) and endogenous melatonin rhythms (37) are especially prominent in youths with depression. In a subgroup of young people with depression, a marked delay in evening melatonin secretion was found to be accompanied by wider temporal circadian disorganization and more severe mood symptoms (38). From this perspective, it may be postulated that the potential of melatonin agonists to alleviate depressive symptoms by improving biological rhythms may be especially high in those young people with marked disturbances in endogenous melatonin rhythms. However, most of the previous agomelatine trials were based on samples covering a wide age range, with a preponderance of middle-aged people.

Converging evidence from both the depression and sleep fields shows enhanced outcomes when pharmacotherapies are combined with psychological or behavioral interventions (39–41). Yet, little is known about the application of such approaches to circadian disturbances in the context of depression. As part of an open-label proof of concept study, we developed a multimodal chronobiological intervention combining: psychoeducation about sleep and circadian rhythms, guided behavioral manipulation of the sleep-wake cycle, and 8 weeks of agomelatine intake. This intervention was implemented as an adjunct to standard clinical care. Focusing on a population known to have high rates of circadian phase delay, we aimed to determine whether mood improvements following this intervention relate to the degree of circadian phase shift.

## Materials and Methods

### Participants

People with unipolar depressive disorders (i.e., major depressive disorder, depression not otherwise specified, or dysthymia) were recruited from early intervention youth services at the clinics of the Brain and Mind Centre in Australia between March 2013 and January 2015. All participants had their first depressive episode before 25 years of age. Diagnoses were confirmed by a mental health professional using DSM-IV criteria and all participants had significant depressive symptoms at study entry (clinician-rated Quick Inventory of Depressive Symptoms, QIDS ≥ 6). None of the participants reported any respiratory, neurological, or other psychiatric disorders (aside from anxiety disorders), had hepatic impairment (i.e., cirrhosis or active liver disease), or had done regular shift-work or transmeridian travel within 60 days prior to study entry. Individuals taking CYP1A2 inhibitors (e.g., fluvoxamine, ciprofloxacin), hypnotics, benzodiazepines, cardiac, or melatonin-based medication were systematically excluded. The dosage of any psychotropic medication had to be stable for at least 2 months prior to study entry.

### Procedures

This study was carried out in accordance with the recommendations of the Human Research Ethics Committee of the University of Sydney. All participants gave written informed consent in accordance with the Declaration of Helsinki. The anonymous dataset analyzed the in current study is available from the corresponding author on reasonable request by independent researchers.

#### Ambulatory Sleep-Wake Monitoring

Participants completed a sleep diary and wore an actigraph (Actiwatch-L/2; Philips Respironics, Bend, USA or GENEActiv, Activinsights, Kimbolton, UK) on the non-dominant wrist for at least a week prior to the intervention start and after 6 weeks of intervention. Actigraphy is a non-invasive tool which objectively measures activity profiles to estimate sleep patterns. All actigraphy data were visually inspected by trained technicians to adjust the start and end of each sleep episode based on information from sleep diaries, event markers and ambient light. A validated algorithm for the Actiwatch data (42) and an equivalent validated algorithm for the GENEActiv data (43) were used to identify periods of wake during the sleep episode with a medium sensitivity threshold of 40 counts per epoch. Sleep onset time, sleep offset time, total sleep time (number of minutes during the sleep period scored as sleep), and sleep efficiency (percentage of the sleep period that was scored as sleep) were averaged over the monitoring period.

#### Circadian Assessment

Before the intervention start and after about 8 weeks of intervention (i.e., at the end of each period of actigraphy monitoring), participants underwent a circadian assessment. Caffeine was prohibited from noon onward on the day of these assessments. The timing of all measurements was based on individual actigraphy-based sleep-wake schedules. Participants were kept under controlled light (< 30 lux), remained seated as much as possible, and were fed temperature controlled snacks at regular times. They were invited to go to sleep 2.5 h after their mean actigraphic sleep onset time and were woken up at their habitual wake time.

Saliva samples were collected with Salivette tubes (Sarstedt, Nümbrecht Germany) every 30 min starting 6.5 h before habitual sleep time until 2 h after habitual sleep onset time. Salivary melatonin (200 μl) was assayed in duplicate by double antibody radioimmunoassay (Buhlmann Laboratories AG, Schönenbuch, Switzerland) with a detection threshold of 0.999 pg/mL (inter-assay coefficient of variation between 8.2 and 15%, intra-assay coefficient: < 10.0% across the standard curve). Dim Light Melatonin Onset (DLMO) was defined by interpolation with a threshold of 3 pg/mL (stable for the three subsequent samples). If melatonin levels did not reach this threshold before the end of the saliva sampling period, DLMO was estimated to occur 30 min after the timing of the last sample. This method may underrate the magnitude of DLMO phase shift (i.e., opposite direction to our hypotheses), but generally preserves the relative order of DLMO timing across participants. To quantify the alignment between endogenous melatonin rhythms and the timing of the sleep-wake cycle, we calculated the time lapse between DLMO and average sleep onset time (DLMO-SleepOn phase angle).

#### Adjunctive Chronobiological Intervention

All participants remained engaged in standard clinical care at the Brain and Mind Centre throughout the study. At the end of the baseline circadian assessment, all participants attended a 1 h psychoeducational session about sleep and circadian rhythms covering the following topics: (i) sleep and circadian education with tailored discussion based on actigraphy data; (ii) individualized plan for progressive phase advance of wake up times; (iii) the role of melatonin in sleep-wake regulation; (iv) a brief overview of the effects and mechanisms of action of agomelatine; and (v) lifestyle factors and behaviors impacting on sleep (e.g., exercise, light, sleep environment, regularity of sleep schedules, foods, stress, anxiety, mood).

Participants then received 8 weeks of open-label pharmacotherapy with agomelatine during which they were asked to progressively shift their wake-up schedule by 15 min each day until they reached their ideal wake time. They were asked to take 25 mg of agomelatine (Valdoxan) each evening, 1–2 h before their individual habitual sleep onset time (except for the night of the follow-up circadian assessment). This dosage was set in accordance with agomelatine's summary of product characteristics and previous efficacy trials for the treatment of major depression. If the participant's care provider deemed that there was no sufficient improvement in depressive symptoms after 4 weeks, this dosage was increased to 50 mg daily. If any new sleep or mood treatment was initiated during the study, participants were automatically excluded from the final dataset.

#### Monitoring and Follow-Up Clinical Assessment

Participants were contacted by telephone on a weekly basis to enquire about treatment adherence and potential side effects. Liver function blood tests were conducted at baseline, as well as, 4, 6, and 12 weeks after the intervention start to monitor hepatic functions (the last time point corresponding to 4 weeks after the end of the intervention). After about 8 weeks of intervention, the was administered again.

### Statistical Analyses

To determine within-participant changes in mood, circadian rhythms and the sleep-wake cycle across the intervention period, paired-samples *t*-tests were used to compare QIDS scores, DLMO, actigraphy parameters and the DLMO-SleepOn phase angle at baseline (before the intervention start) and at follow-up (after 8 weeks of intervention).

Relative change scores for all variables were calculated as the ratio of the value obtained at baseline divided by the value obtained after 8 weeks of intervention (i.e., higher relative change scores reflect higher relative improvements across the intervention period). Pearson correlations were used to assess whether relative change scores on the QIDS were associated with relative change for circadian variables showing significant differences between baseline and follow-up.

## Results

Thirty participants enrolled in this study. Out of these, 2 did not start the study due to abnormal blood results, 2 were withdrawn by the research team due to changes in medications or possible adverse events, and 2 decided to stop the study for reasons unrelated to possible adverse events. Twenty-four participants completed the study: 10 males and 14 females aged between 17 and 28 years old (Mean ± SD: 21.9 ± 3.1 years). On average, depressive symptoms severity at baseline fell within the moderate range (QIDS score range: 9–22; Mean ± SD: 14.7 ± 4.3). Based on QIDS ratings on the week preceding the start of the intervention, 18 (75%) participants reported having a sleep onset latency longer than 30 min at least more than half of the time, 7 (29%) reported multiple nocturnal awakenings of at least 20 min more than half of the time, and 2 (8%) reported waking up at least 1 h before they needed to without being able to fall back to sleep. Twelve (50%) participants were taking other psychotropic medication in addition to agomelatine [antidepressants: *n* = 11 (46%), antipsychotics: *n* = 1 (4%)].

During the intervention phase, 8 participants (33% of the total sample) had an increase in their daily dosage of agomelatine (i.e., from 25 to 50 mg). Across the 8 weeks intervention (i.e., 56 daily doses of agomelatine), 20 participants (83% of the total sample) reported missing no more than 5 doses of agomelatine (i.e., ≤ 9% of the planned doses), and 4 participants (17% of the total sample) reported missing 6–11 doses (i.e., 10–20% of the planned doses). Hepatic functions remained normal across the intervention for all participants. Occurrences of potential adverse events are reported in Table 1. None of these potential adverse events were deemed serious when evaluated by the participants' care providers. Actigraphy data was missing for four participants (17% of the total sample), and melatonin data was missing for five participants (21% of the total sample).

**Table 1:** Possible adverse events.

	**#Participants**	**%Participants**	**Total events**
Difficulty sleeping, sleepiness, or fatigue	12	50.0%	19
Anxiety or agitation	9	37.5%	15
Vivid dreams	9	37.5%	12
Nausea	8	33.3%	11
Headaches	7	29.2%	17
Aggressive behavior	6	25.0%	8
Abdominal swelling or bloating sensation	6	25.0%	10
Back pain	5	20.8%	6
Itchy skin	3	12.5%	3
Suicidal thoughts	3	12.5%	6
Dizziness	2	8.3%	2
Excessive sweating or skin rash	2	8.3%	2
Loss of appetite	2	8.3%	2
Shortness of breath, wheezing, or trouble breathing	2	8.3%	3
Pins and needles sensation on the skin	1	4.2%	2
Blurred vision	1	4.2%	2
Joint pain	1	4.2%	2
Abnormal bleeding or bruising	1	4.2%	1
Swelling of the face, lips, tongue, or body	1	4.2%	1
Discolored urine or stool	0	0.0%	0
Fever	0	0.0%	0
Diarrhea	0	0.0%	0
Yellowing of the skin or eyes	0	0.0%	0
Confusion, loss of consciousness or hallucinations	0	0.0%	0

*Number (#Participants) and percentage (%Participants) of participants who experienced a possible adverse event and total number of possible adverse events (Total Events) across the 56 days of intervention*.

### Changes From Baseline to Follow-Up

**Figure 1:**
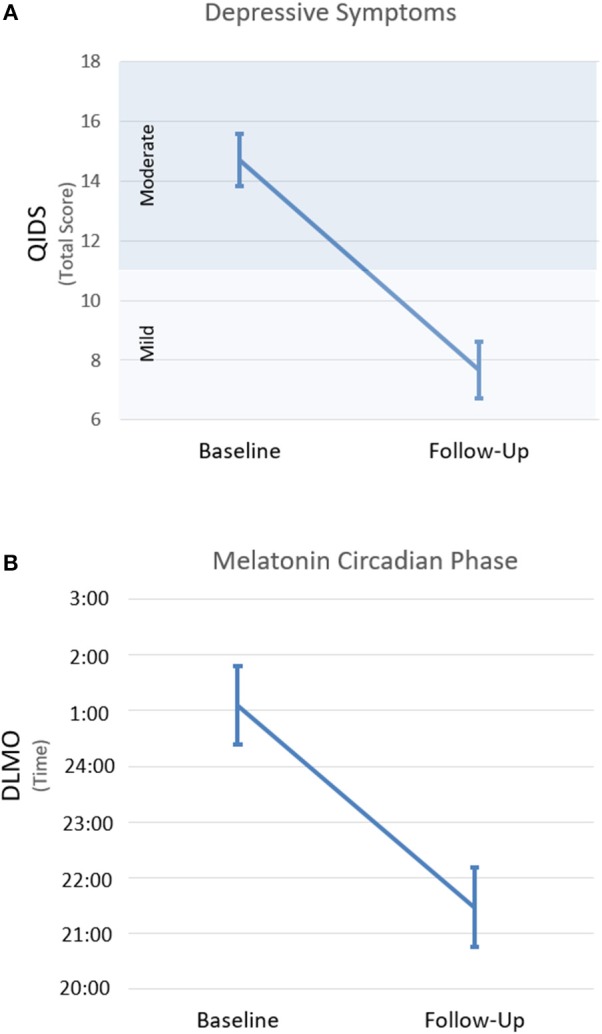
Changes along the course of intervention for depression severity **(A)** and the timing of dim light melatonin onset **(B)**. Changes from baseline (i.e., before intervention start) to follow-up (i.e., after 8 weeks of intervention). Error bars indicate SEM. QIDS, quick inventory of depressive symptoms; DLMO, dim light melatonin onset.

On average, depressive symptoms rated on the QIDS were significantly reduced after 8 weeks of intervention [*n* = 24; *t*_(23)_ = 6.9, *p* < 0.001; Figure 1A]. This difference persisted when total scores were recalculated without the four sleep items [*n* = 24; *t*_(23)_ = 5.9, *p* < 0.001; see Supplementary Figure 1 for individual data]. Based on established clinical thresholds for the QIDS, this represents a mean reduction from moderate to mild depressive symptom severity.

**Figure 2:**
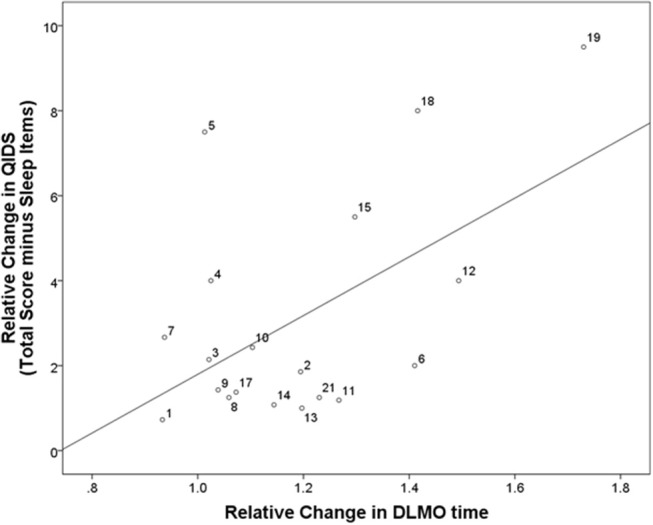
Correlation between relative changes in depressive symptoms and relative changes in DLMO. Correlation between relative changes in depressive symptoms and circadian phase from baseline (i.e., before intervention start) to follow-up (i.e., after 8 weeks of intervention). QIDS, quick inventory of depressive symptoms; DLMO, dim light melatonin onset. Participant identification numbers indicated next to each data point can be matched to Supplementary Figures 1, 2 showing individual raw data at each time point for each participant.

Melatonin did not reach the DLMO threshold at the end of the sampling period (i.e., 2 h past habitual sleep time) for 9 participants at baseline (i.e., Participants 6, 8, 11, 12, 13, 14, 19, 20, 21 in Figure 2) and 2 participants at follow-up (i.e., Participants 8 and 14). Overall, the timing of DLMO was shifted earlier by an average of 3.6 h from baseline to follow-up [*n* = 19; *t*_(18)_ = 4.4, *p* < 0.001; Figure 1B (see Supplementary Figure 2 for individual data)].

Actigraphic sleep estimates at baseline and after 6–8 weeks of intervention are reported in Table 2. On average, from baseline to follow-up, sleep onset time was phase shifted 28 min earlier [*n* = 20; *t*_(19)_ = 2.1, *p* = 0.047] and total sleep time increased by 24 min [*n* = 20; *t*_(19)_ = −2.6, *p* = 0.018]. No significant changes were observed for sleep offset or sleep efficiency. The DLMO-SleepOn phase angle was significantly shorter after compared to before the intervention [*n* = 16; *t*_(15)_ = −3.9, *p* = 0.001].

**Table 2:** Changes in actigraphic sleep estimates.

	**Baseline**		**Follow-up**		***t* (df)**	***p***
	**Mean**	**SD**	**Mean**	**SD**		
Sleep onset	24:37	1:30	24:09	1:15	2.1 (19)	0.047
Sleep offset	9:03	1:21	8:55	1:20	0.5 (19)	0.624
Total sleep time (min)	427.4	51.1	451.2	63.1	−2.6 (19)	0.018
Sleep efficiency (%)	84.6	4.5	85.7	4.7	−1.4 (19)	0.172

*Baseline, before intervention start, Follow-up, after 8 weeks of intervention, SD, standard deviation*.

### Associations Between Circadian and Mood Changes

Relative changes in QIDS scores from baseline to follow-up correlated significantly with relative changes in the timing of DLMO (*n* = 19; *r* = 0.69, *p* = 0.001; Figure 2), and this remained significant when excluding the sleep items of the QIDS (*n* = 19; r = 0.54, *p* = 0.016).

No significant correlation was found between changes in QIDS scores and changes in any of the actigraphic sleep estimates or the DLMO-SleepON phase angle (all *p* > 0.050). When comparing the subgroup of participants taking other psychotropic medications (in addition to agomelatine) to those who were only taking agomelatine, no significant difference in relative changes for any of the depression or circadian measures were observed (all *p* > 0.050).

## Discussion

Based on a proof of concept open-label study, the present findings suggest that 8 weeks of agomelatine intake, provided with psychoeducation and behavioral advice, is accompanied by significant mood improvements in young people with depressive disorders. This was not solely driven by improvements on sleep-related depressive symptoms. On average, based on established clinical thresholds, depressive symptoms dropped from moderate to mild severity during the course of the intervention. Most importantly, while these mood improvements were modest when averaged across the whole sample, the strongest antidepressant responses were observed in those individuals who underwent the largest phase advance in DLMO. This strong correlation between mood and DLMO changes suggests that some of the mechanisms of action underlying the antidepressant effects of this intervention could possibly be linked to the restoration of endogenous melatonin rhythms. To our knowledge, this constitutes the first empirical evidence, in people with non-seasonal depression, that mood improves in parallel with circadian readjustment following a chronobiotic intervention. Supporting the need for larger placebo control trials, these preliminary results reinforce the hypothesis that circadian readjustment may be a relevant target for the treatment of depression in an identifiable subgroup of people: those with later circadian phase.

In this proof-of-concept study, the multimodal aspect of the intervention was designed to optimize clinical outcomes. The 1 h psychoeducation component of the intervention aimed to enhance participant's understanding of their sleep-wake patterns, biological rhythms, potential modulating factors, as well as, the importance of controlling wake up times and the timing of agomelatine intake. We anticipated that this may enhance self-empowerment, healthier sleep/circadian hygiene, and better adherence to the behavioral (phase advancing wake up times) and pharmacological (agomelatine) components of the intervention. Of note, based on current knowledge about psychological approaches to treating sleep disorders, it is unlikely that psychoeducation alone would have driven direct major changes in sleep, circadian rhythms or mood independently of the effects of other active behavioral or pharmacological interventions. In the present study, the behavioral component of the intervention is not likely to have had a significant impact since actigraphy data showed that the intended progressive phase advance of wake-up times did not occur. This lack of significant change in wake up times may be due to the fact that most participants in this sample had to wake up at a fixed time in the morning to attend school or work. Overall, in this specific preliminary study, the pharmacological component of the intervention is likely to have been a primary driver for the observed changes in mood, sleep and circadian rhythms.

Agomelatine has previously been shown to phase shift melatonin in healthy individuals (27), but little was so far known about the effects of this drug on endogenous circadian rhythms in people with depression. While the present findings remain to be replicated in larger placebo-controlled studies, they suggest that youths with depression undergo a marked melatonin phase advance and a shortening in the phase angle between evening melatonin release and sleep initiation following 8 weeks of evening agomelatine intake combined with brief psychoeducational and behavioral interventions. Since DLMO was based on a partial circadian curve, it remains possible that abnormalities in melatonin profiles could also be influenced by overall lower melatonin levels. In any case, the present findings suggest that the intervention led to earlier occurrences of normal levels of melatonin release in the evening. Interestingly, before the intervention, 37% of participants had melatonin levels which still had not reached the DLMO threshold in the 2 h following habitual sleep time. This is in stark contrast with the raise in melatonin typically occurring about 2 h before habitual sleep time in healthy people, suggesting that this subset of participants had a major delay in evening melatonin release with a severe misalignment between melatonin rhythms and the sleep-wake cycle (i.e., inverted phase angle). After the intervention, melatonin did not reach the DLMO threshold within the 2 h following habitual sleep onset time in only 2 of these participants, suggesting that some degree of normalization in evening melatonin secretion took place during the intervention period for most participants.

These changes in melatonin were accompanied by a mild phase advance in sleep onset time and a proportionate lengthening of total sleep duration. Shifting melatonin release earlier in the evening, and/or increasing melatonin levels around bedtime, may have facilitated earlier sleep initiation, thereby extending sleep. This echoes previous findings suggesting that agomelatine improves sleep in people with depression (28, 44–46). The lack of significant changes in sleep efficiency during the intervention period could possibly be due to a ceiling effect. On average, prior to the intervention start, our sample of young participants had an actigraphy-based sleep efficiency of nearly 85%, perhaps leaving little room for improvement.

Despite the positive effects on sleep duration and on the alignment between endogenous melatonin rhythms and the sleep-wake cycle, the antidepressant effects of this multimodal intervention were significantly associated only with the phase advance in DLMO. Thus, the physiological mechanisms of this intervention's mood enhancing effects in youths with depression may rely more heavily on the restoration of endogenous melatonin rhythms than on other sleep factors. We previously found high rates of misalignment between endogenous rhythms and the sleep-wake cycle in young people with depression, and this seemed to be mainly driven by a prominent phase delay in DLMO (38). Consequently, for a large part of that population, adjusting the timing of melatonin release may be a critical factor for the restoration of biological rhythms and the alleviation of depressive symptoms. From this perspective, agomelatine provided with psychoeducation and behavioral advice may trigger the correction of a measurable physiological abnormality known to affect a subgroup of people with depression. This type of treatment thus has the potential to be optimally targeted toward an identifiable subset of people with depression based on distinct pathophysiological profile.

This study has several limitations. The sample size was small and some data was missing. The lack of a placebo condition, and the multidimensional and adjunctive nature of the intervention preclude direct causal inference. Half of the participants were taking other psychotropic medications aside from agomelatine, but these medications were stable for at least 2 months prior to the adjunctive intervention start. Therefore, it is unlikely that the continuous use of these other medications could solely explain all subsequent changes in depressive symptoms (or possible indirect effects of depressive symptoms reduction on sleep or circadian rhythms) during the adjunctive intervention. In addition, there was no significant difference in mood, sleep or circadian outcomes between participants who were taking other psychotropic medications as compared to those who were only taking agomelatine. This strengthens the rationale for investigating chronotherapies as a primary treatment strategy in drug-free participants. Considering the possible synergistic effects of agomelatine on the melatonin and monoamine systems (47), comparative trials with pure melatonin agonists and serotonin antagonists are required. Future comparative studies are also warranted to decipher the potential relative contributions of psychoeducational, behavioral and pharmacological components of multimodal chronobiotic interventions for depression.

While this remains to be replicated in larger placebo-controlled double blind trials, this open-label study in young people with depression provides preliminary evidence suggesting that an adjunctive multimodal chronobiotic intervention combining a psychoeducation session about sleep and circadian rhythms with agomelatine intake in the evening could alleviate depressive symptoms, facilitate earlier secretion of higher levels of melatonin in the evening, and advance the timing of sleep onset time, while extending sleep duration. In this small sample, the degree of phase advance in endogenous evening melatonin release was found to be a significant correlate of mood changes, suggesting that the antidepressant effects of the intervention may possibly be linked to the correction of a measurable physiological abnormality. Overall, this strengthens the hypothesis that chronobiotic interventions may be especially effective to treat depression in people with circadian disruptions. If these findings are replicated, they could offer promising avenues for targeted treatment based on the prior identification of objective pathophysiological phenotypes. Larger studies in more diverse samples are required to evaluate the relationship between initial circadian profile and the pattern of response to chronobiotic interventions.

## Author Contributions

IH, ES, SN, DH, and RR designed the study and wrote the protocol. RR and DB developed the psychoeducational session. JC, K-LF, DW, BW, JS, and RR contributed to study management, data collection, processing and analyses. RR conducted the statistical analysis, and wrote the first draft of the manuscript. All authors reviewed the final manuscript.

### Conflict of Interest Statement

This study was investigator-initiated, and Servier Australia (Hawthorn, Victoria) provided funding support for the study and supplied the medication. The supporters had no role in the design, analysis, interpretation, or publication of this study. IH has been a Commissioner in Australia's National Mental Health Commission since 2012. He is the Co-Director, Health and Policy at the Brain and Mind Centre (BMC) University of Sydney. The BMC operates an early-intervention youth services at Camperdown under contract to headspace. IH has previously led community-based and pharmaceutical industry-supported (Wyeth, Eli Lily, Servier, Pfizer, AstraZeneca) projects focused on the identification and better management of anxiety and depression. He is a Board Member of Psychosis Australia Trust and a member of Veterans Mental Health Clinical Reference group. He is the Chief Scientific Advisor to, and an equity shareholder in, Innowell, a joint venture between the University of Sydney and PwC. DH has received honoraria for educational seminars from Janssen-Cilag and Eli Lilly. The remaining authors declare that the research was conducted in the absence of any commercial or financial relationships that could be construed as a potential conflict of interest.
