# Supplementary material for: Parallel Changes in Mood and Melatonin Rhythm Following an Adjunctive Multimodal Chronobiological Intervention With Agomelatine in People With Depression: A Proof of Concept Open Label Study
Source: Front Psychiatry. 2018 Dec 11;9:624. doi: 10.3389/fpsyt.2018.00624 (PMC6297866; doi:10.3389/fpsyt.2018.00624)
Supplement: Supplementary file 1 [file Data_Sheet_1.docx]

**Supplementary figure 1.** Individual depressive symptoms severity ratings across the intervention period

Baseline: before intervention start, Follow-up: after 8 weeks of intervention, QIDS: Quick Inventory of Depressive Symptoms.

**Supplementary figure 2.** Individual DLMO timing across the intervention period

**
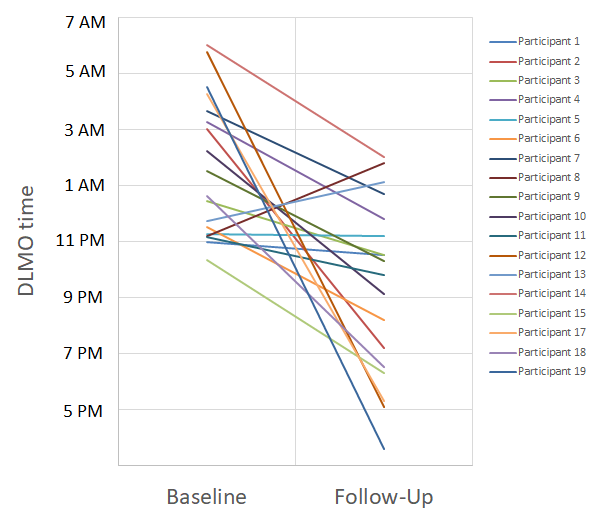
**

Baseline: before intervention start, Follow-up: after 8 weeks of intervention, DLMO: Dim Light Melatonin Onset.
